# Navigating Interstitial Heterotopic Pregnancy: A Case Report

**DOI:** 10.7759/cureus.51854

**Published:** 2024-01-08

**Authors:** Rayhan Karimi, Rachael McCabe

**Affiliations:** 1 Internal Medicine, Edward Via College of Osteopathic Medicine, Spartanburg, USA; 2 Obstetrics and Gynecology, CaroMont Health, Gastonia, USA

**Keywords:** cornual pregnancy, intrauterine pregnancy, ectopic pregnancy, cornuostomy, heterotopic interstitial pregnancy

## Abstract

Interstitial heterotopic pregnancy (IHP), an exceedingly rare and clinically intricate phenomenon, presents a diagnosis that challenges healthcare providers in obstetric care. This case report provides valuable insights into the complexities of diagnosing and managing IHPs, shedding light on the pivotal role of early and precise identification. Through a meticulous examination of a 28-year-old patient's clinical history and diagnostic journey, this report underscores the significance of advanced imaging techniques and swift decision making, ultimately leading to the accurate diagnosis of an IHP. Furthermore, it highlights the life-saving importance of cornuostomy as a safe and effective intervention, preserving the intrauterine pregnancy while successfully resolving the ectopic gestation. This case report serves as a compelling reminder of the critical need for timely diagnosis and individualized treatment in cases of IHPs, ultimately enhancing the understanding and management of this rare obstetric condition.

## Introduction

Interstitial heterotopic pregnancies (IHPs), characterized by the condition in which one embryo implants in the interstitial part of the fallopian tube, also known as the cornual region, while another embryo implants within the uterine cavity, are a medical rarity. Oron and Tulandi found the incidence of heterotopic pregnancies in the general population to be one in 30,000 pregnancies, with IHPs accounting for only a 3-4% of this fraction [1], displaying the need for additional research and guidelines for treatment options. 

The uterus is anatomically divided into several distinct regions, each playing a specific role in gestation. The cornua, or uterine horns, represent the uppermost part of the uterus where the fallopian tubes meet, forming a triangle-like structure. This region is particularly vulnerable to the occurrence of IHPs. In the context of IHPs, the fertilized egg erroneously implants within the cornua, leading to the development of an ectopic pregnancy. The cornua have a unique vascular supply, and their anatomical characteristics make them predisposed to challenges during surgical interventions, including hemorrhage and future pregnancy viability.

Historically, the diagnosis of IHPs has posed a considerable clinical dilemma because patients often present with symptoms and signs indicative of a normal intrauterine pregnancy (IUP), including a positive beta-human chorionic gonadotropin (β-hCG) and typical early pregnancy ultrasound findings. The ectopic component, residing within the cornua, remains inconspicuous, and as Condous et al. have studied, it can go undetected until a rupture occurs, potentially leading to catastrophic hemorrhage [2].

Recent advances in imaging techniques, particularly transvaginal ultrasound with high-resolution capabilities, have enhanced our ability to diagnose cornual heterotopic pregnancies earlier in gestation. However, the optimal management strategy remains a topic of debate. The need to preserve the IUP while safely addressing the ectopic gestation poses a delicate clinical challenge as physicians must also factor in maternal mortality and future fertility concerns. 

In cases of IHP, where the risk of rupture in the corneal region can be as high as 49% [3], and maternal mortality rates range from 2% to 3%, selecting the most appropriate treatment approach requires a thorough understanding of the nuances associated with each option. The available treatment modalities are cornual resection, methotrexate injection, expectant management, and cornuostomy. Cornual wedge resection stands as the conventional surgical approach for IHPs. However, due to the anatomical intricacies of the cornua, resection is associated with an elevated risk of hemorrhage, blood loss, and uterine rupture. In hemodynamically unstable patients, surgical intervention is recommended, with the caveat of potential intrauterine fetal loss, necessitating thorough patient consent. Medical management involves the injection of methotrexate or potassium chloride (KCl) into the ectopic pregnancy, guided by ultrasound. Medical management introduces the risk of intrauterine leak and the potential for congenital abnormalities, necessitating careful consideration and patient counseling [4].

Expectant management involves a watchful waiting approach without immediate intervention. This strategy carries the risk of ectopic rupture, leading to significant blood loss. The potential consequences encompass intrauterine fetal loss and, critically, maternal mortality, making this option a delicate balance between cautious observation and the need for timely intervention. Lastly, cornuostomy represents a distinct surgical technique tailored for IHPs. Cornuostomy is considered as an alternative surgical approach with potential advantages over traditional cornual resection with a less invasive approach.

Hemorrhagic complications often arise from surgical interventions or inherent vascular complexities, which necessitate innovative approaches for effective control. Epinephrine, a powerful adrenergic agonist, has demonstrated utility in various medical contexts for its vasoconstrictive properties. In the context of hemorrhage control in heterotopic pregnancies, the use of epinephrine presents a compelling avenue. By inducing vasoconstriction, epinephrine has the potential to mitigate blood loss, particularly in the delicate vascular environment of the cornua, where IHPs commonly occur. Another option that is commonly used in surgeries is vasopressin. Vasopressin acts on vascular smooth muscle receptors, causing intense vasoconstriction in blood vessels. This effect is particularly beneficial in minimizing intraoperative bleeding and enhancing the surgeon's ability to visualize structures in the surgical field.

As IHPs gain recognition in contemporary clinical practice, it is imperative to consolidate our understanding of their clinical presentation, diagnostic distinctions, and optimal management strategies. In this case report, we present an exploration of this patient's clinical course, diagnostic journey, and therapeutic decisions, and aim to contribute to the growing body of knowledge surrounding this condition.

## Case presentation

**Figure 1:**
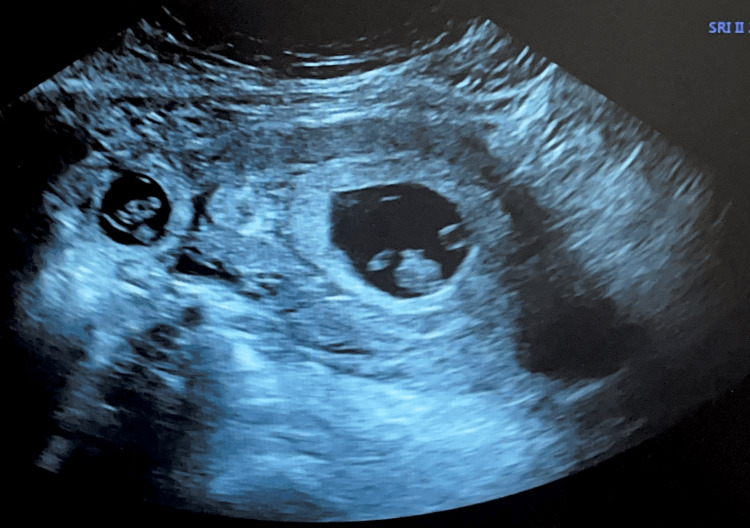
Transvaginal ultrasonography image of the uterus (transverse view) showing an intrauterine gestation with a crown rump length of 2.10 cm (right) coexisting with an ectopic tubal pregnancy with a crown rump length of 1.53 cm (left).

We present a case of a 28-year-old G3P1011 with a history of essential hypertension, who was incidentally diagnosed with an interstitial heterotopic pregnancy during a routine confirmation of pregnancy visit. Upon evaluation of the ultrasound in clinic, it revealed two concurrent pregnancies - an intrauterine pregnancy and an ectopic pregnancy within the right interstitial tube. Ultrasound noted diamniotic, dichorionic twins corresponding with an eight-week gestational age, both with cardiac activity, as seen in Figure 1.

**Figure 2:**
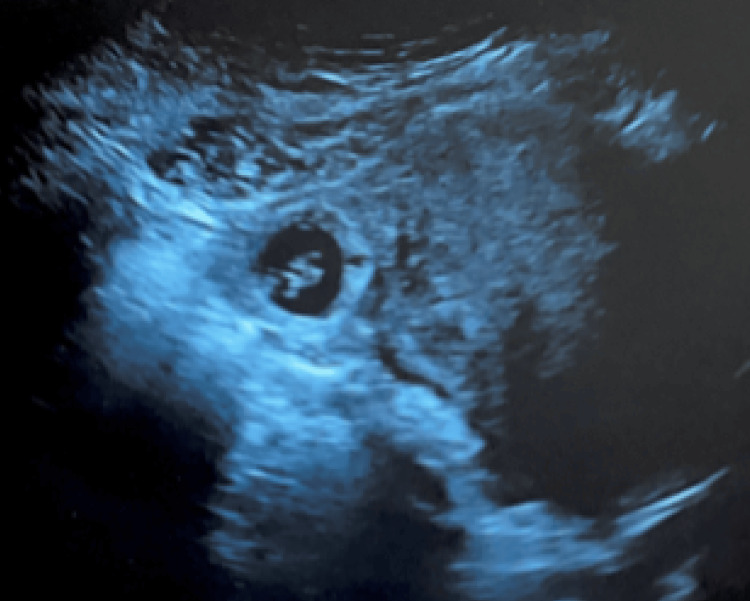
Transvaginal ultrasonography image of the uterus (transverse view) showing the ectopic pregnancy, presumably in the ampulla of the fallopian tube.

The patient remained asymptomatic; however, she was immediately referred to a high-risk obstetric clinic for further investigation. There was question regarding the location of the ectopic, and it was presumed to be in the typical ectopic location, the ampulla of the fallopian tube, as seen in the ultrasound image in Figure 2*.*

**Figure 3:**
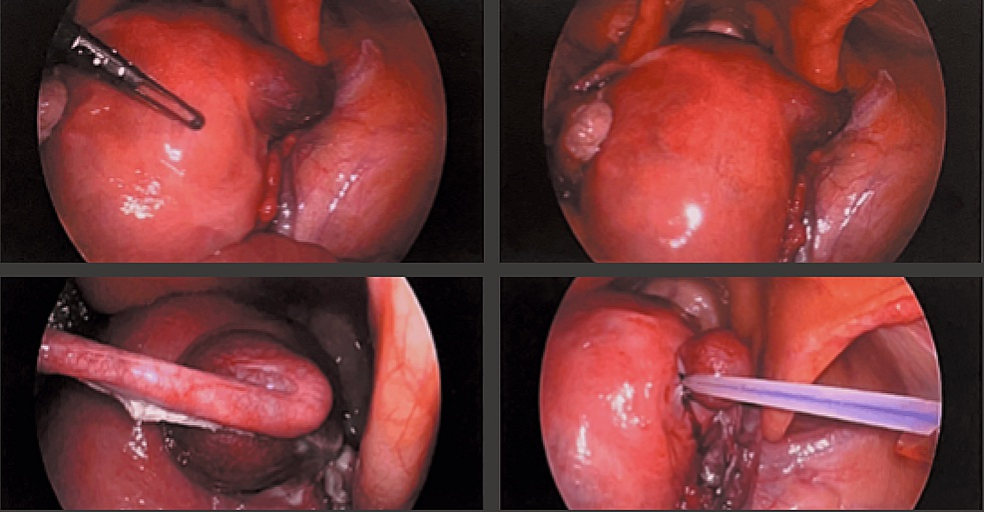
Diagnostic laparoscopic images showing the uterus and the cornual location of the ectopic pregnancy

Given the potential risks associated with this condition, the patient was promptly transferred from clinic to the operating room for surgical intervention. General anesthesia was implemented without difficulty and the patient was placed in the dorsal lithotomy position. Diagnostic laparoscopy was performed to visualize the uterus and associated structures. After visualization, there was concern that the ectopic was not fully in the ampulla of the fallopian tube, but rather in the cornua of the uterus (Figure 3).

Therefore, an intraoperative phone consult with the high-risk obstetrics physician was executed, and it was determined that the location and size of the ectopic posed too great of risk to leave in situ. The surgery team began the procedure to remove the ectopic. A LigaSure Vessel Sealing device was used to cut and saucer across the mesosalpinx and detach the tube up to the location of the ectopic. A PDS Endoloop Ethicon was used to keep the uterus away from LigaSure. The tube containing parts of the ectopic were released. Subsequently, bleeding was not able to be controlled by LigaSure due to extensive vascularization of the ectopic implantation site, leading to sudden hemorrhage. 

**Figure 4:**
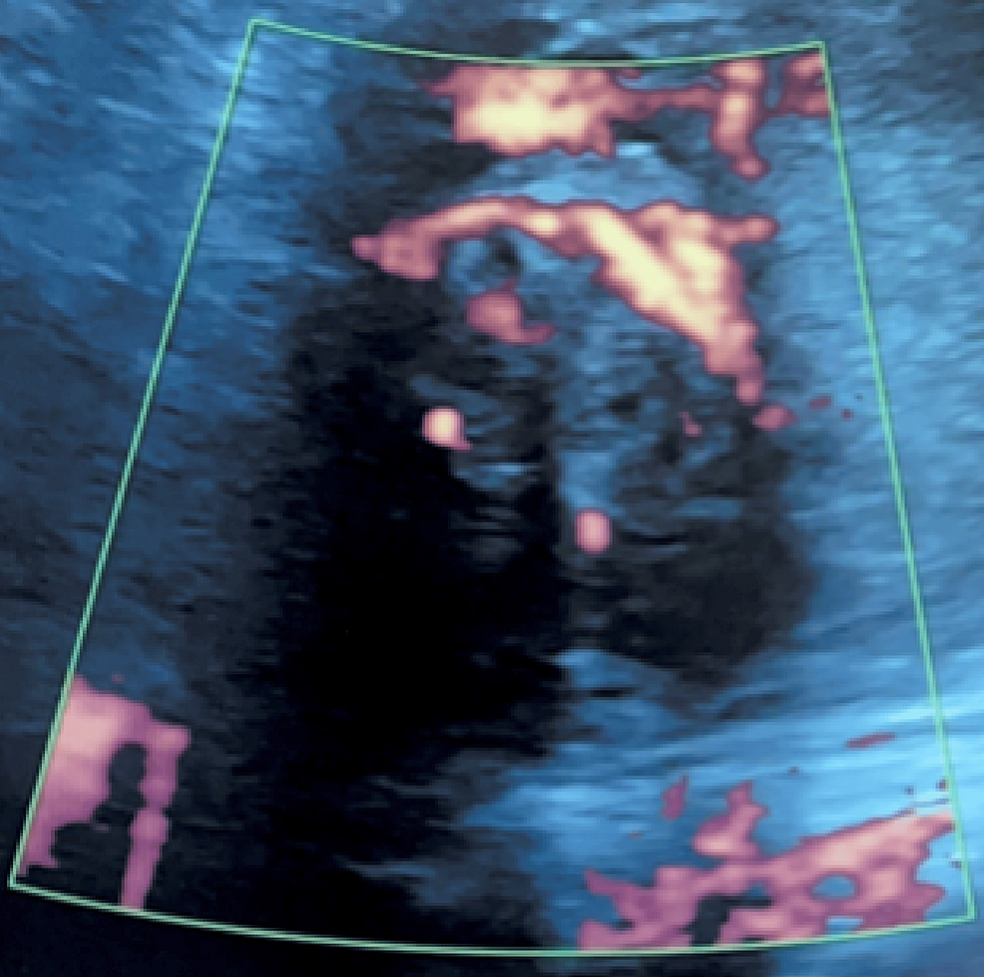
Ultrasound images of the uterus (transverse view) displaying a central cystic area with circumferential blood flow with no yolk sac or fetal pole.

In response to the critical situation, a shift in the surgical approach was swiftly executed. The surgical team performed laparotomy and made a horizontal incision 2 cm above the pubic symphysis. Next, to achieve hemostasis, implementation of Kelly clamps on the bleeding right cornua was enacted. An incision was made into the right cornua, and a surgical spoon was employed to effectively extract the ectopic products. The surgery team completed right cornuostomy, right salpingectomy, and subsequent repair of the right cornua. Upon evaluation of the right cornua, there was no evidence of ballooning or clear margins of the ectopic. It was to be noted that further dissection would put the IUP at risk and compromise the myometrium. VICRYL sutures were used to perform a Heaney tie below the Kelly clamps. This adapted strategy not only addressed the immediate bleeding concern but also aimed to preserve the viability of the intrauterine pregnancy. Postoperatively, the patient experienced a nonconsequential 3 cm broad ligament hematoma that was discovered on MRI and treated conservatively with pain management and observation (Figure 4). The patient progressed to deliver a healthy infant via cesarean section at 36 weeks.

## Discussion

Treatment options

The management of IHPs involves several treatment options, each with its own advantages and considerations. Expectant management may be considered in cases of early IHP, where the risk of tubal rupture is perceived as low. However, this approach carries the inherent risk of tubal rupture and severe hemorrhage, especially in cases with advanced gestational age or significant fetal cardiac activity. Medical therapy using methotrexate, a folic acid antagonist, has been employed in IHPs, particularly when the patient is hemodynamically stable, and the ectopic pregnancy is unruptured. However, methotrexate injections can also lead to tubal rupture, occurring in 7-14% of patients with an ectopic pregnancy. In addition, as Leziak et al. stated, side effects, such as hepatotoxicity, myelosuppression, and stomatitis, occur in about 37% of women [5]. Surgical options, such as laparoscopy and laparotomy, offer a more definitive solution for IHP management. Studies by D'Ambrosio et al. show that surgery does not increase the risk of maternal or fetal adverse events and surgery possesses the favorable traits of quicker postoperative recovery and return to regular activities [6]. Furthermore, cornuostomy, a minimally invasive surgical technique involving the removal of the ectopic gestational sac through a small incision in the cornual region, has emerged as a promising alternative. Cornuostomy combines the advantages of surgical management with a reduced risk of uterine damage and quicker recovery, making it an attractive option for select cases [7]. Further research and larger studies are needed to establish clear guidelines for the management of IHPs and to refine our understanding of the most appropriate approach for optimizing outcomes in these rare but high-risk cases.

Case reflection

The use of epinephrine in surgical procedures during IUPs is a subject of clinical consideration, as it involves balancing the need for hemostasis and surgical control with potential effects on maternal and fetal well-being. Epinephrine is a potent vasoconstrictor, which can temporarily reduce blood flow to the uterine arteries and the placenta when used locally to control bleeding. However, studies and clinical experience have suggested that, when administered in controlled and monitored conditions, the effects of epinephrine on IUPs are generally well tolerated. One study by Ngan et al. investigated the effects of vasopressors, including epinephrine, on intrauterine pressure and fetal circulation during gynecological laparoscopy in pregnant women. The study concluded that although vasopressors caused temporary alterations in intrauterine pressure and fetal circulation, these effects were not associated with adverse fetal outcomes, indicating that the use of vasopressors like epinephrine can be safe under appropriate conditions [8]. In addition, in a review study using 41 patients with IHPs, 82% of them used diluted intramyometrial vasopressin at the beginning of the operation to minimize blood loss and increase visibility [9]. Therefore, it can be concluded that the judicial use of vasopressin can be used in the cornua in cases of IHPs. It is important to note that the safety of vasopressin or epinephrine during pregnancy depends on factors, such as the gestational age of the fetus, the specific surgical procedure, the concentration, the dosage used, and the overall health of the mother. During the laparoscopy in this patient, an Endoloop and LigaSure were used to remove the ectopic pregnancy. However, there was hemorrhaging from the ectopic due to extensive vascularization. In reflection of this case, the implementation of epinephrine or vasopressin could have mitigated the risk of hemorrhage and also given better visualization of structures. Due to the lack of research and familiarity of IHPs, the surgery team was not comfortable using epinephrine or vasopressin close to the IUP and therefore was not used during surgery. 

Recommendations

In review of this case, recommendations to future clinicians would be to begin treatment for IHPs with laparoscopic cornuostomy, and if signs of hemodynamic instability or hemorrhage occur, a rapid conversion to laparotomy should be implemented. One study analyzed 17 cases of IHPs and showed that compared to laparotomy, laparoscopic cornual resection had shorter hospital stay, decreased operative time, and decreased blood loss [10]. Translating these data to the more minimally invasive route of cornuostomy, these statistics should be heightened. However, while laparoscopy is often preferred for its minimally invasive nature, the risk of encountering significant bleeding and the need for rapid intervention in ectopics should not be underestimated. This patient course suggests that in cases of IHP with complex vascularization or high-risk features, such as advanced gestational age or active bleeding, early conversion to laparotomy may be prudent to achieve better control over the surgical field and minimize the risk of hemorrhage and maternal mortality. In order to decrease risk of maternal mortality, clinicians should begin the surgical approach with laparoscopy and should switch to laparotomy intraoperatively when there is hemodynamic instability or uncontrollable hemorrhage.

Furthermore, after a review of the literature, it can be determined that the use of epinephrine and vasopressin are safe to use in cases of IHPs; therefore, it is recommended that clinicians should implement them for their vasoconstrictive properties and ability to minimize blood loss during the operation.

## Conclusions

The successful management of this IHP through cornuostomy underscores the importance of early detection and prompt intervention in complex ectopic pregnancies. This case report highlights the feasibility of cornuostomy as a valuable surgical approach in preserving the IUP while effectively managing the ectopic component. By achieving a favorable outcome for the IUP, this case serves as a basis for cornuostomy and can be considered high priority in terms of treatment guidelines in the future for surgeons. Cornusotomy reduces extensive damage to the uterus for future pregnancies and decreases the risk of uterine rupture during delivery. As we continue to advance our understanding of these complex pregnancies, the lessons learned from this case will contribute to improved care and decision-making for similar cases in the future.
